# Guided self-help cognitive behavioral intervention for VoicEs (GiVE): study protocol for a pilot randomized controlled trial

**DOI:** 10.1186/s13063-016-1494-y

**Published:** 2016-07-26

**Authors:** Cassie M. Hazell, Mark Hayward, Kate Cavanagh, Anna-Marie Jones, Clara Strauss

**Affiliations:** 1 School of Psychology, University of Sussex, Falmer, Brighton, BN1 9QJ UK; 2 R&D Department, Sussex Partnership NHS Foundation Trust, Sussex Education Centre, Hove, BN3 7HZ UK

**Keywords:** Voices, Auditory hallucinations, Psychosis, Cognitive behavioral therapy, CBT, Low intensity, Self-help

## Abstract

**Background:**

Cognitive behavior therapy for psychosis (CBTp) is an effective intervention for people who hear distressing voices (auditory hallucinations). However, there continues to be a problem of poor access to CBTp. Constraints on health care funding require this problem to be addressed without a substantial increase in funding. One solution is to develop guided self-help forms of CBTp to improve access, and a symptom-specific focus on, for example, distressing voices (auditory verbal hallucinations) has the potential to enhance effectiveness. We term this *cognitive behavior therapy for distressing voices* (CBTv).

**Methods/design:**

This trial is an external pilot randomized controlled trial comparing the effects of 12-week guided self-help CBTv (with eight therapist support sessions) with a wait list control condition. Informed consent will be obtained from each participant. Half of the 30 participants will be randomized to receive guided self-help CBTv immediately; the remaining half will receive the intervention after a 12-week delay. All participants will continue with their usual treatment throughout the study. Outcomes will be assessed using questionnaires completed at baseline and 12 weeks postrandomization. Interviews will be offered to all those who receive therapy immediately to explore their experiences with the intervention.

**Discussion:**

The outcomes of this trial, both quantitative and qualitative, will inform the design of a definitive randomized controlled trial of guided self-help CBTv. If this intervention is effective, it could help to increase access to CBT for those who hear distressing voices.

**Trial registration:**

ISRCTN registration number ISRCTN77762753. Registered on 23 July 2015.

## Background

Hearing voices (i.e., auditory verbal hallucinations) is a common experience [1]; some people find this experience very distressing and hear voices in the context of a mental health problem [2]. Hearing distressing voices is a symptom of psychotic spectrum disorders, such as schizophrenia [3], but voices can also be present in the context of other mental health conditions, such as borderline personality disorder [4], dissociative identity disorder [5], bipolar disorder [6], and posttraumatic stress disorder [7].

The authors of a meta-analysis found cognitive behavioral therapy for psychosis (CBTp) to be an effective treatment for distressing voices [8]. National treatment guidelines issued in a number of countries recommend that everyone with a psychotic disorder be offered CBTp [9–11], with the guidelines stating that at least 16 individual sessions of CBTp should be offered.

Despite evidence for its effectiveness and recommendations in national treatment guidelines, CBTp is not widely available. Even though the United Kingdom has led the way in promoting the implementation of CBTp [12], the authors of a report by the U.K. Schizophrenia Commission [13] found only 10 % of people with psychosis were offered CBTp. More recent figures suggest this rate may have actually decreased [14].

The most consistently reported barrier to implementation is insufficient resources to meet the demand, including both insufficient numbers of therapists trained in providing CBTp [15] and trained therapists’ lack of protected time [14]. The current economic climate has had a deleterious impact on mental health funding across the globe [16]. Consequently, it is not realistic to expect that resources required to deliver CBTp in line with treatment guidelines will be available in the near future. An alternative approach is to use available resources more efficiently. This could include offering CBTp self-help resources with a therapist’s guidance, an approach that requires less therapist resources than standard interventions [17]. We propose a guided self-help form of CBTp that could be a suitable intervention for this client group. Our approach requires less therapeutic contact time (8 sessions) than the recommended 16 sessions, so the same number of therapists could, in principle, offer an intervention to twice as many people over the course of a year.

The authors of multiple meta-analyses have found that guided self-help cognitive behavioral therapy (CBT) is effective for treatment of anxiety and depression [18, 19], and, as a consequence, guided self-help CBT is offered routinely in the United Kingdom for people experiencing these difficulties [20]. Evidence is emerging that briefer forms of CBTp may also be effective. A meta-analysis found that, in comparison to control conditions, briefer forms of CBTp (i.e., trials offering fewer than the recommended 16 therapy sessions) significantly improved psychosis symptoms at posttherapy and during follow-up [21]. However, the authors of a previous meta-analysis of CBTp more broadly showed that trial quality is an important moderator of treatment effects; with high-quality CBTp trials producing smaller effect sizes than lower-quality trials [22, 23]. Therefore, lower-quality trials may overestimate the effectiveness of briefer forms of CBTp.

In addition, in an effort to improve the effectiveness of CBTp, the field is moving away from generic CBTp for the broad range of psychotic symptoms and toward symptom-specific CBTp, with recent trials being focused on CBTp for delusions [24] and distressing voices [25]. However, to our knowledge, there has been no research into the potential of guided self-help for distressing voices, and this is the aim of the present study.

This study is an external pilot randomized controlled trial (RCT) being conducted prior to a definitive trial of guided self-help CBTp for people distressed by hearing voices (which we term *CBTv*). Guided self-help CBTv aims to reduce the distress associated with the experience of hearing voices. Therefore, the primary hypothesis for the trial will be that, compared with the delayed therapy control group, those who receive guided self-help CBTv will experience a reduction in the distress associated with voices. Secondary hypotheses will be that those receiving guided self-help CBTv will report improvement in negative and positive beliefs about voices, self-esteem, and assertive relating in comparison with the control group, which are the mechanisms through which the therapy is proposed to work [26].

The following are the aims of the present pilot study: (1) to determine whether a full trial is justified, (2) to establish the effect size on the primary outcome (voice-related distress) for a sample size calculation for a definitive trial, and (3) to address questions concerning study recruitment, retention, and acceptability. Specifically, between-group treatment effects will determine whether a definitive RCT of guided self-help CBTv is justified; that is, we will proceed if the effect size on the primary outcome is in favor of CBTv in comparison to the delayed therapy control condition and if the 95 % CI for this effect contains the minimal clinically important difference (MCID). If so, this study will provide the parameters needed to estimate the sample size for a definitive RCT. Finally, we will assess whether the study design is acceptable to participants using recruitment and retention rates and qualitative interviews with participants. Both study dropout and therapy dropout rates will be reported with a view to understanding reasons for dropout and developing strategies to minimize dropout in a definitive trial.

## Methods/design

This study is a pragmatic, single-blind, external pilot RCT with two parallel arms and 1:1 allocation. A rater blinded to allocation will conduct postintervention assessments. A total of 30 participants will be recruited; half will be randomly allocated to receive guided self-help CBTv immediately (immediate therapy), and half will join a wait list for the same intervention (delayed therapy). Both groups will maintain their usual mental health care throughout the course of the study.

**Fig. 1:**
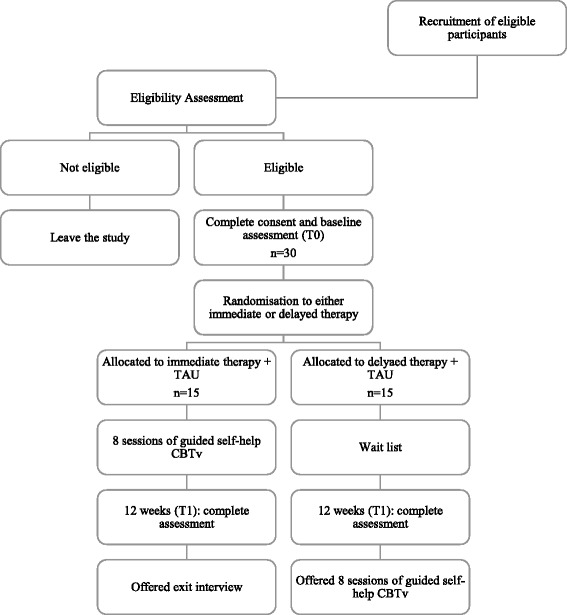
Consolidated Standards of Reporting Trials (CONSORT) diagram of participant progression through the Guided self-help cognitive behavioral intervention for VoicEs trial. *CBTv* cognitive behavioral therapy for voices, *TAU* treatment as usual

The randomization of participants will be conducted by an independent statistician, using a 1:1 ratio random permuted block randomization (with block sizes of two, four, and six). The research team will be blinded to the block size. Figure 1 illustrates the process of randomization and group allocation.

The outcome data will be collected at time 0 (before randomization), and at time 1 (12 weeks postrandomization). Time 1 assessments will be conducted by a research assistant blinded to the participant’s group allocation. All of the participants allocated to receive immediate therapy will be asked to complete an exit interview to discuss their experience with the therapy.

To assess adherence to the protocol, all therapists will be asked to complete a checklist after each session with each client, indicating the elements of the protocol they have covered. The checklist has been designed specifically for this intervention.

### Participants

Participants will be service users recruited from an NHS mental health trust in the South of England. Clinicians will be asked to refer eligible service users from among their caseload. Potential participants will also be identified using a database of people who have agreed to be contacted about research studies.

In line with the recommendations of Julious [27] for pilot RCTs, 12 participants per arm are required for this pilot RCT. The authors of a meta-analysis of briefer forms of CBTp found the maximum dropout rate to be 13.5 % [21]. Using this as a guideline, we have overestimated a potential attrition rate of 20 % for the present study, meaning a total of 30 participants will be recruited (15 per arm).

The inclusion criteria for participants are that they must (1) be aged 18 years or older; (2) be distressed by hearing voices; which will be operationalized by requiring participants to score at least 3 out of 5 on item 5 (How much do the voices interfere with your daily activities?), 6 (How distressing are the voices that you hear?), or 7 (How bad do the voices make you feel about yourself) on the Hamilton Program for Schizophrenic Voices Questionnaire (HPSVQ) [28], equivalent to moderate levels of interference, distress, and impact, respectively; (3) have heard voices for the at least 1 year; (4) are not currently in, or have plans to receive, another psychological intervention during the course of the study; and (5) are able to read and write in English at the level required for the self-help course.

Participants will be excluded from the trial if they have a primary diagnosis of substance misuse or if an organic illness is determined as the reason for hearing voices. Otherwise, diagnosis is not an inclusion or exclusion criterion, given that distressing voices are common across a range of diagnoses (i.e., schizophrenia [3], borderline personality disorder [4], dissociative identity disorder [5], bipolar disorder [6], posttraumatic stress disorder [7]).

### Planned intervention

The 12-week intervention is based on and guided by the self-help book *Overcoming Distressing Voices* [26] and an accompanying workbook that summarizes the key chapters. The therapists and participants will have the same therapy materials (book and workbook). Therapist guidance will be offered over a maximum of 8 sessions (30–60 minutes per session) delivered over the 12 weeks. The intervention will be guided by clinical psychologists with expertise in CBTp, and participants will be encouraged to engage with the structured self-help workbook throughout. The intervention protocol has been developed in collaboration with clinicians and people with lived experience of hearing voices.

The intervention consists of five modules: (1) The Coping module will look at ways of managing voice distress; current coping strategies will be evaluated, and new ones considered; (2) the Me module will address self-esteem; negative beliefs about the self will be re-evaluated, and positive beliefs will be strengthened; (3) the My Voices module will target unhelpful beliefs that are typically associated with voices, focusing on re-evaluating these beliefs; (4) the My Relationships module will give the participant an opportunity to work on a difficult relationship (including the relationship with voices), the aim being to develop more assertive ways of relating; and (5) the Looking to the Future module offers an opportunity for reflection and develops a plan for taking learning from the self-help course forward into the future.

Participants will keep the book and workbook after therapy has ended, providing a resource they can return to at a later date. Participants will be free to drop out of the intervention at any point. Therapists will each receive training in the intervention from CS and MH (authors of the self-help book), and monthly therapist group supervision will be offered.

### Wait-list control (delayed therapy)

Participants allocated to delayed therapy will join a waiting list for the intervention. The waiting period will be approximately 12 weeks after randomization, at which point participants will receive the intervention as outlined above.

Throughout the trial (including the waiting period), participants will continue with their usual mental health care. This will likely involve taking psychiatric medication and having regular contact with a clinician. The type of medication participants are taking, as well as any changes in these across the span of the study, will be recorded.

### Measures

The primary outcome will be the HPSVQ voice impact subscale [28]. The HPSVQ voice impact subscale has five items rated on a 5-point (0–4) scale and measures the level of distress and impact that voices have on the person. The phenomenology subscale (four items) will also be included in this trial (and will be a secondary measure in the definitive trial). The questionnaire contains nine items in total and has strong concurrent validity (all *r* > 0.80) as well as good internal consistency (all α > 0.82) [29].

The following measures of secondary outcomes are also included in this trial:

1. The Choice of Outcome in CBT for Psychoses questionnaire [30] was developed in partnership with service users. It has 22 items measuring service user-defined recovery. Two of the items give participants the opportunity to include their own recovery goals for CBT. The severity scale of this questionnaire has a high level of test-retest reliability (α = 0.83).
2. The Hospital Anxiety and Depression Scale (HADS) [31] measures clinical levels of anxiety and/or depression. The HADS has 12 items. The English version of this questionnaire has good internal consistency (α = 0.80) [32].
3. The Short Warwick-Edinburgh Mental Well-being Scale (SWEMBS) [33] uses seven items to measure psychological well-being. The SWEMBS has demonstrated good reliability (α = 0.91).
4. The Rosenberg Self-Esteem Scale (RSES) [34] consists of ten items. It was originally developed to measure self-esteem among adolescents, but it has since been used with adults with strong psychometric properties. The RSES has strong levels of test-retest reliability over time (*r* = 0.85).
5. The Brief Core Schema Scale (BCSS) self-scale [35] measures participants’ endorsement of positive and negative beliefs about themselves. The BCSS self-scale has 12 items and has strong internal consistency when given to clinical participants (α = 0.84).
6. The Person’s Relating to Others Questionnaire short version (PROQ3) [36] measures social relating (person to person) in terms of their proximity and power. The questionnaire includes 48 items, and all scales show acceptable levels of internal consistency (all scales α > 0.70).
7. The Voice and You [37] measures the same relational dimensions as the PROQ3 [36] but in the context of the participant’s relationship with their voices (hearer to voice). The scale has 28 items and demonstrates good internal consistency across all of the scales (all scales α > 0.80).
8. The Beliefs about Voices Questionnaire–Revised [38] has 35 items and measures the strength of a range of beliefs about voices. All scales have acceptable levels of internal consistency (all scales α > 0.74).

Sessional measures will be taken using six visual analogue scale questions, with each question targeting an intended therapy outcome or mechanism. An exit interview will be administered postintervention to participants in the immediate therapy condition. The Change Interview [39] will be used at that time to ask participants to consider any changes they have experienced over the course of the intervention and, if so, what they attribute these changes to. Helpful, unhelpful, and missing aspects of the intervention are also explored within the Change Interview. The Change Interview also asks participants to provide feedback on their experience with the research study itself, as well as to provide suggestions for future research in the area. The exit interview will also include additional questions on participants’ experiences of the study process (e.g., assessments, consent, randomization) [40].

### Data collection and storage

All data will be collected and stored in line with the Data Protection Act (1998). Assessments will be completed using tablet computers so that data are entered and stored electronically. Anonymized data will be stored on password-protected computers and will be available to members of the research team. Any files that contain personal information of participants will be kept in a password-protected electronic file or in locked filing cabinets on NHS or university sites. To maximize study retention, participants will be invited to complete postintervention assessments even if they have dropped out of the intervention, and all assessments will be conducted at a time and place most convenient to the participant.

### Planned analysis

All of the analyses will be carried out once data collection has been completed. IBM SPSS Statistics version 22 software (IBM, Armonk, NY, USA) will be used to conduct all quantitative analyses. The findings of this trial will be reported using the Consolidated Standards of Reporting Trials (CONSORT) guidelines. The recruitment rates for this trial will be reported as a ratio of the number of potential participants approached to the number who consent to participate. The retention rates will be reported first as the percentage of participants who drop out of the study (i.e., do not complete time 1 assessments) and second as the percentage of participants who drop out of therapy before exposure (receive fewer than four sessions). If study or therapy dropout rates are greater than 33 %, this will indicate that changes to the study and/or therapy protocols may be needed. The acceptability of the study and therapy will be assessed using retention rates and feedback obtained through the exit interview.

The analysis for this trial will be mostly descriptive. We will report the means and/or medians (as appropriate) as well as SD and minimum and maximum scores at time 0 and time 1 for both groups on primary and secondary outcomes. Participants’ characteristics will be reported using frequency counts and percentages, including the characteristics of any participants who drop out of the study and/or therapy. Analysis of covariance will be used to estimate T1 between-group differences on primary and secondary measures, controlling for baseline scores. Ninety-five percent confidence intervals will be calculated. Effect sizes will be reported as Cohen’s *d* (unstandardized effect size divided by the baseline pooled SD). We will look at the effect sizes using both an intention-to-treat analysis approach and a per-protocol analysis (looking only at participants who attended at least 50 % of therapy sessions). A definitive trial will be considered to be justified if there is a between-group effect in favor of CBTv on the primary outcome (HPSVQ voice impact subscale [28]) and if the 95 % CI contains the MCID of 2 points in favor of CBTv.

The exit interviews will be transcribed into NVivo software files (QSR International, Doncaster, Australia) and analyzed using thematic analysis in accordance with the Braun and Clarke [41] protocol. The aim of the analysis will be to identify common patterns across interviews that reflect the participants’ experiences of therapy and of the study processes. These themes, as well as the quantitative results, will be used to refine the study and therapy protocols. If a definitive trial is justified, then the power calculations will be based upon the between-groups effect size on the HPSVQ voice impact subscale [28].

### Research governance

**Fig. 2:**
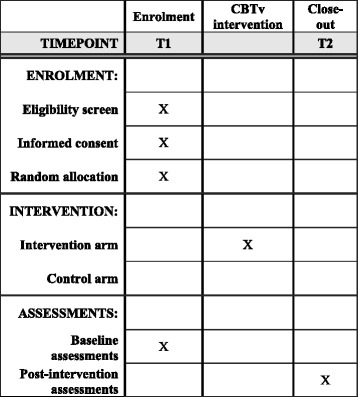
Schedule of enrollment, intervention, and assessments. *CBTv* cognitive behavioral therapy for voices

This protocol has been prepared in line with the Standard Protocol Items: Recommendations for Interventional Trials (SPIRIT) guidelines [42, 43]. See Fig. 2  and additional material for a copy of the SPIRIT figure and checklist for this study.

The trial is sponsored by the University of Sussex and funded through a doctoral studentship award given to CMH by the Economic Social and Research Council (ES/J500173/1) and Sussex Partnership NHS Foundation Trust. Ethics approval has been granted by the NHS Health Research Authority North West – Lancaster Research Ethics Committee (REC) (15/NW/0575). Written informed consent will be obtained from each participant by the lead author. A copy of the participant information sheet and consent form can be obtained from the corresponding author.

A Trial Steering Committee (TSC) will be established in accordance with Medical Research Council guidelines [44] and will monitor the conduct of the trial and adherence to the study protocol. The TSC will include an independent chair, independent experts, and lay members. If necessary, applications to make significant protocol amendments will be made to the REC and study sponsor. A Data Monitoring Committee is not deemed necessary, given the pilot nature of the current study.

The U.K. National Institute of Health Research Good Clinical Practice guidelines will be followed, which includes reporting and investigation of any adverse events during the study. In line with these guidelines, the research team will ensure that the patient care team members are aware of any adverse events. If an adverse event is deemed to be related to the study, it will be reported to the study sponsor. The sponsor will then determine whether the study needs to be stopped and if an investigation of the adverse event is needed.

### Dissemination

Findings will be written up for publication in an open-access, peer-reviewed journal, and a lay summary of findings will be sent to all study participants.

## Discussion

The development of guided self-help CBTv is one approach that could potentially address the problem of limited access to CBT in the United Kingdom for people who experience psychosis [45]. However, it would be inadvisable to offer guided self-help CBTv routinely, as we do not yet know if it is acceptable or effective at reducing voice-related distress; it is an approach that widens access but that, if ineffective, would be of little use and a waste of limited resources.

This external pilot RCT will generate effect sizes of CBTv in comparison to the wait-list control group on the primary outcome (voice-related distress) and on secondary measures. The trial results will also indicate recruitment and retention rates and intervention acceptability. These parameters will inform the design and sample size calculation for a definitive trial. If found to be effective within a definitive trial, guided self-help CBTv has the potential to widen access to an effective, evidence-based intervention for people distressed by hearing voices.

### Trial status

The trial has received ethical and governance approvals. Recruitment began in September 2015 and is planned to end in January 2016.

## Abbreviations

BCSS, Brief Core Schema Scale; CBT, cognitive behavioral therapy; CBTp, cognitive behavioral therapy for psychosis; CBTv, cognitive behavioral therapy for voices; CONSORT, Consolidated Standards of Reporting Trials; GiVE, Guided self-help cognitive behavioral intervention for VoicEs; HADS, Hospital Anxiety and Depression Scale; HPSVQ, Hamilton Program for Schizophrenic Voices Questionnaire; MCID, minimal clinically important difference; PROQ3, Person’s Relating to Others Questionnaire short version; RCT, randomized controlled trial; REC, Research Ethics Committee; RSES, Rosenberg Self-Esteem Scale; SPIRIT, Standard Protocol Items: Recommendations for Interventional Trials; SWEMBS, Short Warwick-Edinburgh Mental Well-being Scale; TAU, treatment as usual; TSC, Trial Steering Committee
